# BiCuSeO Thermoelectrics: An Update on Recent Progress and Perspective

**DOI:** 10.3390/ma10020198

**Published:** 2017-02-17

**Authors:** Xiaoxuan Zhang, Cheng Chang, Yiming Zhou, Li-Dong Zhao

**Affiliations:** School of Materials Science and Engineering, Beihang University, Beijing 100191, China; zhang1346795@126.com (X.Z.); ccbuaa523@163.com (C.C.); aliex_7@163.com (Y.Z.)

**Keywords:** BiCuSeO, thermoelectric, electrical conductivity, Seebeck coefficient, thermal conductivity

## Abstract

A BiCuSeO system has been reported as a promising thermoelectric material and has attracted great attention in the thermoelectric community since 2010. Recently, several remarkable studies have been reported and the *ZT* of BiCuSeO was pushed to a higher level. It motivates us to systematically summarize the recent reports on the BiCuSeO system. In this short review, we start with several attempts to optimize thermoelectric properties of BiCuSeO. Then, we introduce several opinions to explore the origins of low thermal conductivity for BiCuSeO. Several approaches to enhance thermoelectric performance are also summarized, including modulation doping, introducing dual-vacancies, and dual-doping, etc. At last, we propose some possible strategies for enhancing thermoelectric performance of BiCuSeO in future research.

## 1. Introduction

Thermoelectric (TE) materials have been widely developed to provide a direct solution for energy conversion between electricity and heat. As one of the most promising thermoelectric systems, the BiCuSeO family was first reported in 2010 [1] and has attracted ever-increasing attention in recent years [2,3,4,5,6,7,8,9,10].

BiCuSeO crystallizes in a layered ZrCuSiAs structure type with space group *P4*/*nmm* [11]. The crystal structure of BiCuSeO is formed by fluorite (Bi_2_O_2_)^2+^ layers and anti-fluorite (Cu_2_O_2_)^2−^ layers which stacking along the *c*-axis. (Bi_2_O_2_)^2+^ layers act as carrier reservoir while (Cu_2_O_2_)^2−^ layers are responsible for transporting carriers [1]. Pristine BiCuSeO is a *p*-type semiconductor, in which holes are generated from either Cu- or Bi-vacancies which participate in transportation in the conductive (Cu_2_O_2_)^2−^ layers. Due to its layered structure, BiCuSeO shows anisotropic transport behaviors along in-plane and out-plane directions for both electrical and thermal conductivity. The electrical and the thermal transport properties along the in-plane direction outperform those along the out-plane direction. However, the Seebeck coefficient is isotropic [7].

Compared with the state-of-the-art TE materials including lead and tin chalcogenides [12,13,14,15,16,17,18], Bi_2_Te_3_-based systems [19,20,21,22,23], skutterudites [24,25,26,27,28,29], etc. BiCuSeO features lower-cost and non-toxic elements and better thermal stability in the medium temperature range. Additionally, due to the large Seebeck coefficient, low thermal conductivity and relatively low electrical conductivity, the dimensionless thermoelectric figure-of-merit (*ZT*) value of the BiCuSeO system has boosted to the maximum *ZT* of 1.5 [10], from the initial 0.76 for Sr-doped samples [1], in the past six years. The obvious enhancement in *ZT* subverts the general opinions that thermoelectric oxides usually have low *ZT* values due to poor electrical conductivity and high thermal conductivity.

Since the review paper summarized by Zhao et al. in 2014 [11], a lot of new achievements have been obtained in BiCuSeO system, which motivate us to update these results to show the current progress in BiCuSeO system. In this review, we summarize the latest attempts and methods on optimizing thermoelectric properties of BiCuSeO system, and then introduce several opinions on the origins of low thermal conductivity. Additionally, some typically promising approaches including modulation doping [8], dual-vacancies [9] and dual-doping [10] are summarized. In the end, we propose some outlooks and perspectives for future research.

## 2. A Short Review for Various Attempts to Optimize BiCuSeO System

### 2.1. Enhancing Thermoelectric Properties through Doping and Compositing

It is well known that the thermal conductivity of BiCuSeO is impressively low. Thus, most recent attention has focused on improving the electrical transport properties through various attempting approaches. For instance, considering the carrier concentration of undoped BiCuSeO is as low as ~1 × 10^18^ cm^−3^ [11], the most general approach is to enhance the carrier concentration by doping on Bi sites or Cu sites. Indeed, the carrier concentration was increased by alkaline earth elements M^2+^ (M = Mg, Ca, Sr, and Ba) doping [1,2,4,30]. Besides these alkaline-earth elements, Ag, Na, La, Zn, Cd, and S doping in BiCuSeO [31,32,33,34,35,36,37,38] were reported and exhibited potential performance in medium temperature range. In addition, the compositing with high electrical conductive phases (La_0.8_Sr_0.2_CoO_3_ [39], Cu_2_Se [40]) could successfully improve the thermoelectric properties of pristine BiCuSeO through enhancing the electrical conductivity.

### 2.2. Developing Less Time-Consuming Methods

Initially, it was suggested that polycrystalline BiCuSeO compounds should be synthesized through a multi-step solid-state reaction (SSR), which is complicated and time-consuming. Therefore, any facile or time-saving methods are advanced for future scale-up of resultant products. Ren et al. [41] explored the synthesis of polycrystalline Bi_1−*x*_Pb*_x_*CuSeO through an ultrafast self-propagating high-temperature synthesis (SHS) method. In addition, Yang et al. [42] adopted SHS to synthesize polycrystalline Bi_0.94_Pb_0.06_CuSeO and further explored the thermodynamic reaction mechanism in details. It was confirmed that the entire reaction process consists of four parts. External heating initiated two rapid SHS reactions (2Bi + 3Se = Bi_2_Se_3_, 2Cu + Se = Cu_2_Se) which emitted heat and drove the other two reactions (Bi_2_Se_3_ + 2Bi_2_O_3_ = 3Bi_2_SeO_2_, 3Bi_2_SeO_2_ + 3Cu_2_Se = 6BiCuSeO). Through optimizing reaction parameters such as sample density and reaction atmosphere, etc. the *ZT* value could be enhanced to 1.2 at 923 K for Bi_0.94_Pb_0.06_CuSeO [43].

Although the mechanical alloying (MA) method was applied to BiCuSeO systems in 2013 [32], it was not widely used because of the uncontrollable Bi_2_O_3_ impurity. Compared with solid-state reaction, mechanical alloying is simple, less time-consuming, and much more convenient for large-scale productions. Taking into account these advantages, Wu et al. systematically investigated the mechanical alloying mechanism for BiCuSeO [33], instead of raw materials (Bi, Bi_2_O_3_, Cu, and Se) powders used in the solid-state reaction, Bi, CuO, and Se were selected as starting powders to avoid the Bi_2_O_3_ second phase. Encouragingly, pure single phase BiCuSeO was obtained through ball milling at 500 rpm for 7 h. Apparently, the above results confirmed that mechanical alloying is an effective and promising method to synthesize BiCuSeO [8,24].

### 2.3. Attempting to Synthesize BiCuSeO Single Crystals

In BiCuSeO systems, due to the layered structure, carrier scattering along the in-plane direction is much smaller than that along the out-plane direction. Therefore, we can expect a much higher carrier mobility in the single crystal than that of 22 cm^2^·V^−1^·s^−1^ obtained in polycrystalline BiCuSeO [11], hence a considerable *ZT* value. To date, it is still a challenge to synthesize large size BiCuSeO single crystal. Dong et al. [44] made a significant step in BiCuSeO single crystal synthesis. They successfully synthesized BiCuSeO single crystals through a flux method at 963 K, 1003 K and 1048 K, respectively. BiCuSeO crystals with a typical size of 2.5 × 2 × 0.05 mm^3^ were obtained [45]. Meanwhile, Wu et al. [46] synthesized Bi_1−*x*_Pb*_x_*CuSeO film with high texture orientation through the pulsed laser deposition technique on the SrTiO_3_ (001) substrates, a high power factor of ~1.2 mW·m^−1^·K^−2^ at 673 K was realized. Therefore, a higher *ZT* can be expected in BiCuSeO single crystals. However, the challenge still lies in obtaining sufficiently large single crystals to investigate the thermal transport properties.

Apart from various attempts to enhance thermoelectric performance mentioned above, the interested readers are encouraged to refer to these extensive works that have been done on BiCuSeO systems, such as ultrathin BiCuSeO nanosheets [47], elastic and thermal properties [48,49], phonon transport [50,51], oxidation states [52], Cu vacancies [53,54], thermal stability [55,56], and the TEM study on BiCuSO and BiCuSeO that shows that Cu deficiency also plays a role in the stability and properties of those layered oxychalcogenides [54]. All of these studies largely enrich the understanding of BiCuSeO systems and have a great sense for future research.

## 3. Exploring the Origins of Low Thermal Conductivity in BiCuSeO

The large *ZT* value of BiCuSeO is derived from its low thermal conductivity (κ) in temperatures ranging from 300 K (~0.9 W·m^−1^·K^−1^) to 923 K (~0.45 W·m^−1^·K^−1^) [6]. The low thermal conductivity of BiCuSeO system is supposed to originate from its strong crystal anharmonicity (larger Grüneisen parameter, γ ~ 1.5) [11], weak chemical bond (Young’s modulus, E ~ 78.8 GPa at 300 K) [3], and low Debye temperature (Θ_D_ ~ 243 K) [11]. Thus, any further detailed investigations on its thermal conductivity mechanism will help readers to clarify the low thermal transport behaviors. Here, we introduce several of the latest studies that may reveal the origins of low thermal conductivity for BiCuSeO.

### 3.1. Low Thermal Conductivity Due to In-Layer/Interlayer Anharmonic Vibrations

Ding et al. [57] systematically studied the vibration modes of BiCuSeO and proved that the anharmonic vibrations and structural scattering of phonons are partially caused by in-layer and interlayer off-phase vibration modes. Apart from this, the calculated Grüneisen parameters indicate that Bi is the source of strong crystal anharmonicity.

**Figure 1:**
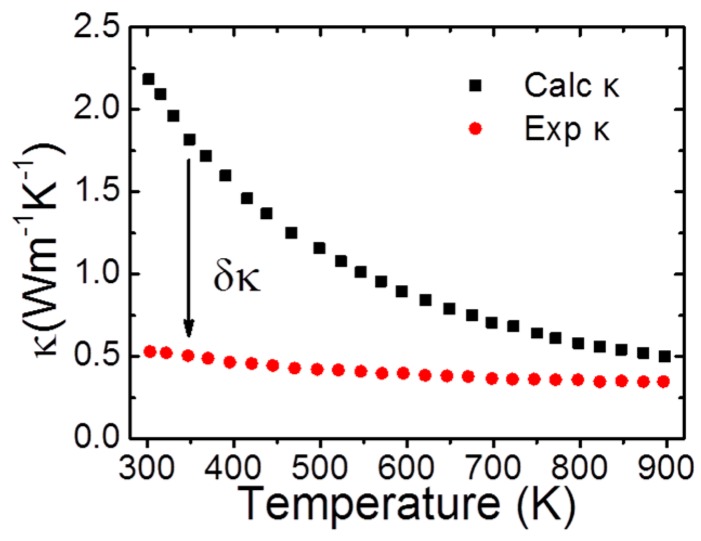
Calculated thermal conductivity. The black squares represent the calculated total thermal conductivity, and the red circles represent the experimental data. Reproduced with permission from Reference [57]. Copyright 2015, IOP Publishing.

Figure 1 shows the thermal conductivity of BiCuSeO. Given a defect-free BiCuSeO crystal, both calculated values and experimental values are almost fitted at high temperatures. However, at low temperatures, the difference between two lines is very significant. To explain the origin of this difference, it is necessary to analyze the phonon spectrum and the power spectrum (PS) of velocity autocorrelation function (VAF) for BiCuSeO. Through analyzing the vibration modes of each branch and studying the atomic motions, an unexpected movement was found. The Cu-Se atom layer vibrates synchronously and the Bi-O atom layer vibrates with a different phase. This discovery directly indicates the existence of interlayer interaction.

**Figure 2:**
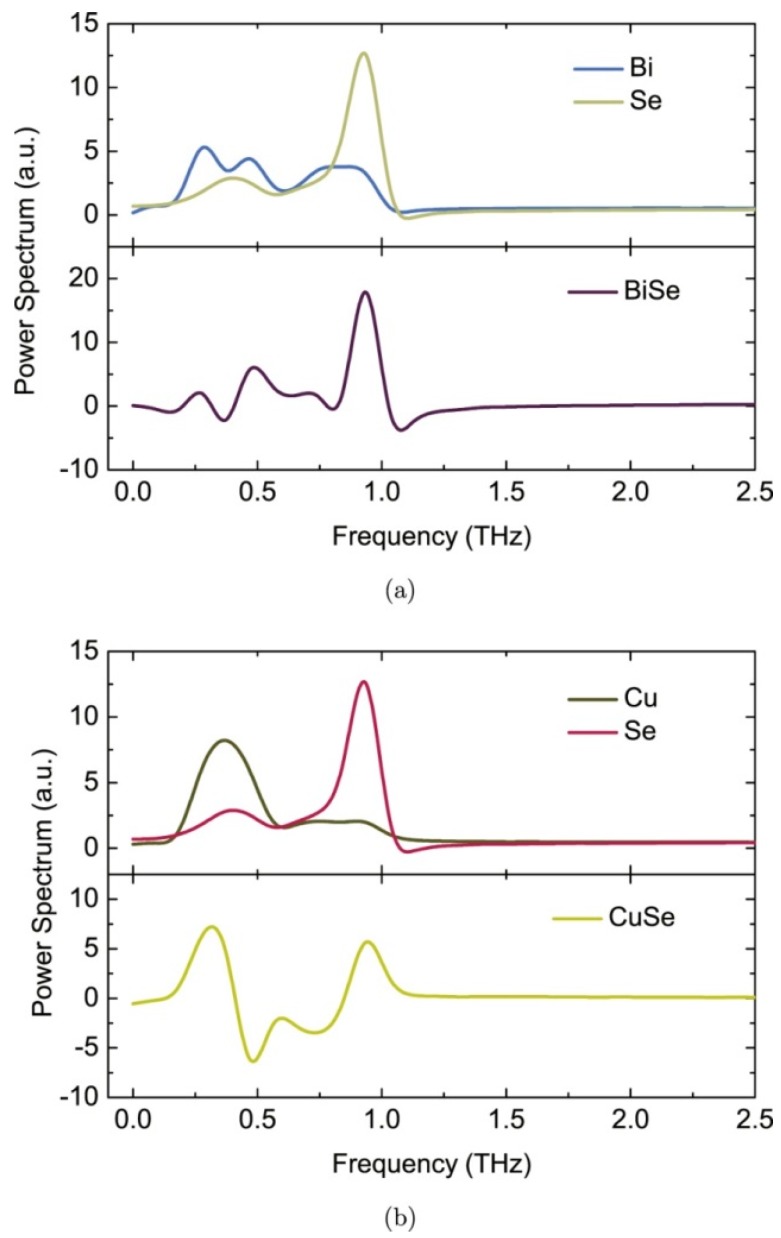
Power spectrum of the self-and cross-VAF. (**a**) Power spectrum of self- and cross-VAF of Bi and Se. Blue and beige lines represent Bi and Se self-VAF, respectively. The Bi/Se cross-VAF shows with purple line; (**b**) Power spectrum of self- and cross-VAF of Cu and Se. Brown and purple lines represent Cu and Se self-VAF, respectively. The Cu/Se cross-VAF is represented yellow line. Reproduced with permission from Reference [46]. Copyright 2015, IOP Publishing.

Figure 2 shows the calculated power spectrum (PS) of the velocity autocorrelation function (VAF). From the PS, the frequencies of different atomic motions are determined. As shown in Figure 2a, if the vibration frequencies of Bi and Se are synchronous, their peaks in the PS of self-VAF will appear at the same frequencies in the cross-VAF spectrum [57]. It is readily seen from the Figure 2a that the negative peaks in the PS of Bi/Se at 0.37 and 0.84 do not exist in the PS of Bi or Se. This means that the vibration frequencies between Bi and Se are different, and proves the existence of interlayer off-phase interaction by comparing with the above results. Similar to that shown in Figure 2a, negative peaks in the PS of Cu/Se can be found at 0.47 and 0.75 THz in Figure 2b, confirming the in-layer interaction between Cu and Se.

**Figure 3:**
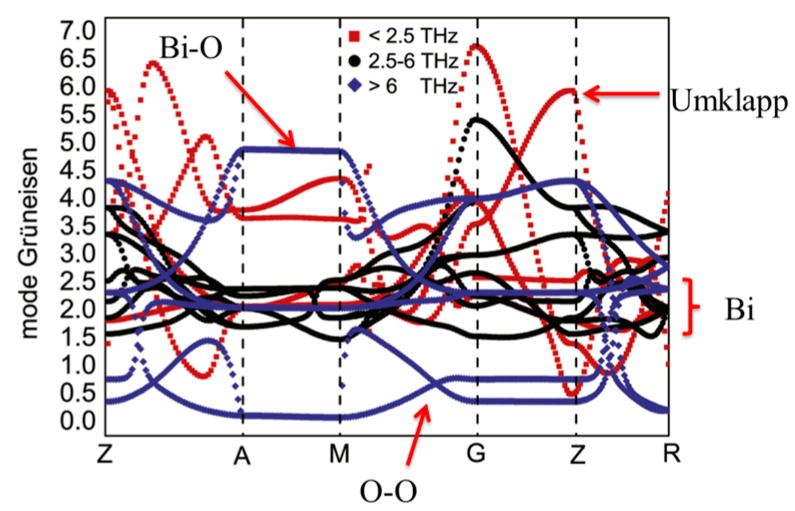
Mode Grüneisen parameters of BiCuSeO along the high symmetry path Z-A-M-G-Z-R-A. The mode Grüneisen parameters of acoustic branches are shown with red squares, black circles are related to the frequencies between 2.5 and 6 THz, and the phonon modes of O above 6 THz are shown with blue diamonds. Reproduced with permission from Reference [46]. Copyright 2015 IOP Publishing.

The mode Grüneisen parameters were calculated by quasi-harmonic approximation. As shown in Figure 3, at low frequency (<2.5 THz), due to the strong interaction between acoustic phonons and optical phonons, acoustic phonons can be significantly scattered by the phonon–phonon Umklapp processes. At middle frequency (2.5–6 THz), the mode Grüneisen parameters from approximately 2 to 2.5 are contributed by the heavy element Bi around the G and Z points. Considering that the O is light and the O–O bonds are mainly involved to the Grüneisen parameters below 1, the large mode Grüneisen parameters above 6THz are actually related to Bi–O bonds. Thus, the viewpoint that large Grüneisen parameter of BiCuSeO almost entirely related to heavy Bi was confirmed by their calculations. Overall, the low thermal conductivity was confirmed to derive from the in-layer and interlayer off-phase vibration modes and heavy Bi.

### 3.2. Heavy Bi Is a Predominant Factor That Causes Low Thermal Conductivity

**Figure 4:**
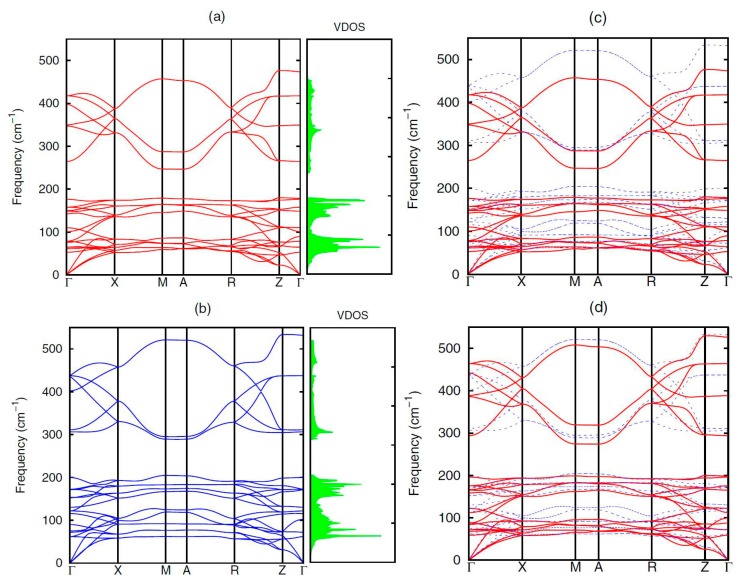
Red line and blue line in (**a**,**b**) represent the calculated phonon dispersion of BiCuSeO and LaCuSeO along the high-symmetry lines of the Brillouin zone; (**c**) Calculated phonon dispersion curves of BiCuSeO (red) and LaCuSeO (blue) for a direct comparison; (**d**) Red solid lines represent the normalized phonon dispersion data of BiCuSeO (**a**), while the blue dashed lines represent the calculated original phonon dispersion data of LaCuSeO. Reproduced with permission from Reference [47]. Copyright 2015, The American Physical Society.

Saha [58] carried out the first-principles density functional theory (DFT) calculation to compare the lattice dynamics, phonon dispersion, and Mode Grüneisen dispersion between BiCuSeO and LaCuSeO. It was found that the better thermal insulator of BiCuSeO than that of LaCuSeO is likely due to the mass difference between Bi and La. Figure 4 shows the calculated phonon dispersion and DOS of (a) BiCuSeO and (b) LaCuSeO along the high-symmetry lines of the Brillouin zone. For the low-frequency modes, because of the same structure and stable interatomic force (IF) constants, the mode frequency difference of BiCuSeO and LaCuSeO can be described by the mass difference. Figure 4c puts the calculated phonon dispersion of BiCuSeO and LaCuSeO into one picture for a direct comparison. In Figure 4d, it can be seen that the red solid lines and the blue dashed lines fit very well in the low-frequency acoustic region, which means the difference between BiCuSeO and LaCuSeO at low-frequency acoustic phonons mode is caused by atomic mass discrepancies between Bi and La.

**Figure 5:**
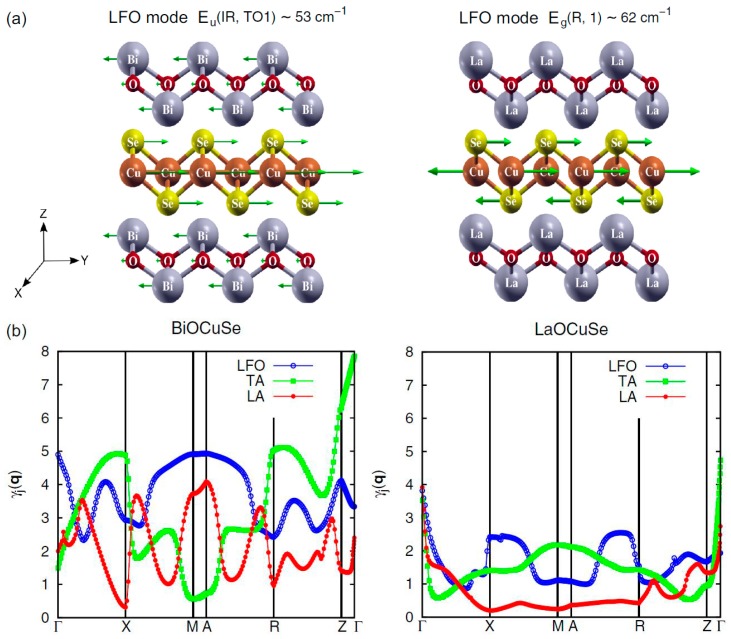
(**a**) Atomic displacement patterns *Eu* (IR,TO1) ~ 53 cm^−1^ and *Eg* (R,1) ~ 62 cm^−1^ for the respective lowest-frequency optical (LFO) mode in BiCuSeO (**left**) and in LaCuSeO (**right**), arrows represent the atomic movement directions; (**b**) Mode Grüneisen dispersion of the above LFO mode (blue) in comparison to that of the longitudinal acoustic (red) and transverse acoustic (green) movement in BiCuSeO (**left**) and in LaCuSeO (**right**), respectively. Reproduced with permission from Reference [47]. Copyright 2015, The American Physical Society.

Figure 5a shows the atomic displacement patterns for the lowest-frequency optical (LFO) mode of BiCuSeO (*Eu* (IR,TO1) ~ 53 cm^−1^) and LaCuSeO (*Eg* (R,1) ~ 62 cm^−1^). The larger displacement in heavy Bi atoms is observed, which leads to higher anharmonic effects. Figure 5b shows the mode Grüneisen dispersion of above lowest-frequency optical (LFO), longitudinal acoustic (LA), and transverse acoustic (TA) modes in BiCuSeO and LaCuSeO. Because LFO mode *Eu* (IR,TO1) in BiCuSeO behaves as a quasi-acoustic mode [58], the acoustic modes can be hybridized more strongly in BiCuSeO than in LaCuSeO. It can lead to an extensive acoustic-optical phonon scattering and then low thermal conductivity. Overall, the same structure as they are, heavy Bi outperforms La in reducing the thermal conductivity (κ). It illustrates the contribution of the heavy Bi element to the low thermal conductivity in BiCuSeO. In 2016, Saha et al. [48] used first-principle calculations to continuously explore the origins of low thermal conductivity of BiCuSeO. Compared with LaCuSeO, they found that BiCuSeO has a lower shear modulus, lower Young’s modulus, lower acoustic velocity, lower Debye temperature, and larger Grüneisen parameter.

### 3.3. Dose Cu Matter in Low Thermal Conductivity?

**Figure 6:**
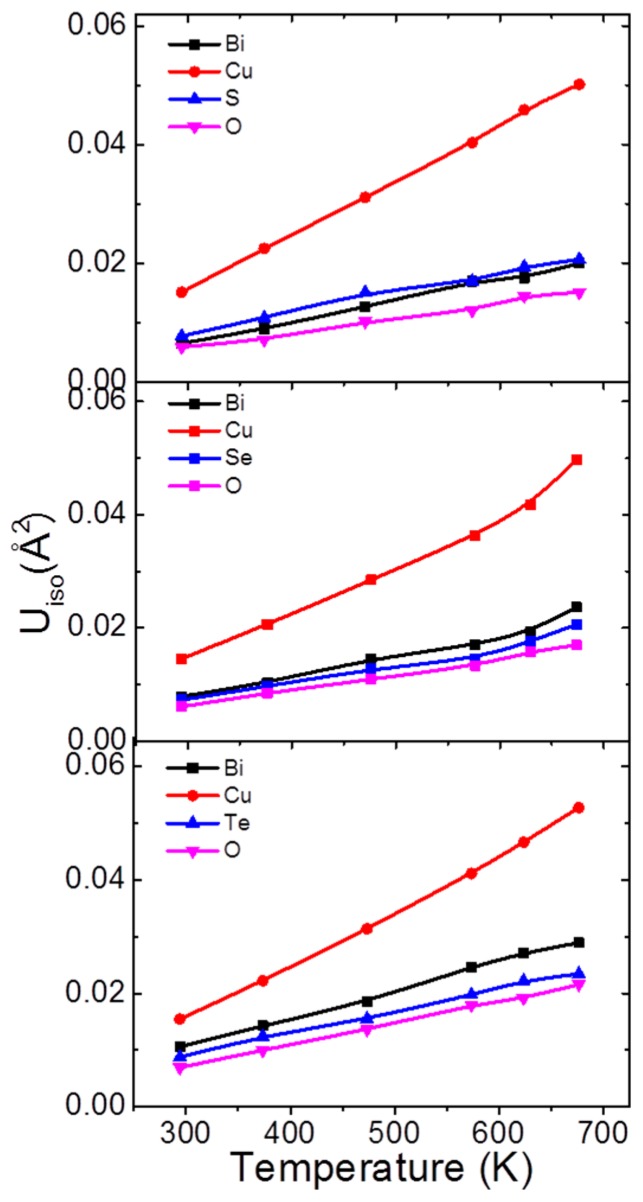
ADPs for BiCuMO (M = S, Se, Te) as a function of temperature. Reproduced with permission from Reference [48]. Copyright 2015, The American Royal Society of Chemitry.

Vaqueiro et al. [59] believe the origins of low thermal conductivity of BiCuSeO mostly comes from Cu atoms and partly from Bi atoms. Through first principle calculation and in-situ neutron diffraction experiments, a low-energy vibrational mode of Cu in BiCuSeO was unveiled. Powder neutron diffraction data on BiCuMO (M = S, Se, Te) revealed the atomic displacement parameter (ADP) among the atoms. As shown in Figure 6, in all the three samples (BiCuMO, M = S, Se, Te), the ADP for Cu is persistently larger than those for other elements in the whole temperature range. It illustrates that Cu possesses a stronger local mobility.

**Figure 7:**
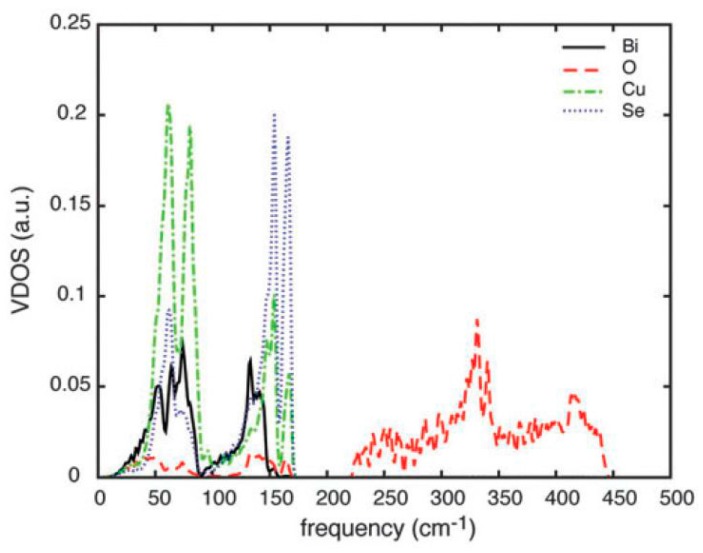
Total vibrational DOS of BiCuSeO projected on each element (Bi, Cu, Se, O). Reproduced with permission from Reference [48]. Copyright 2015, The American Royal Society of Chemitry.

Phonon vibrations for BiCuSeO were also calculated in order to explore the origins of the low thermal conductivity. As shown in Figure 7, the total vibrational DOS of BiCuSeO is projected on each element. At low frequency, the VDOS of Cu is much higher than that of Bi, which indicates that the Cu has a greater effect on total vibration than Bi for BiCuSeO. Meanwhile, through calculating the mode-resolved Grüneisen parameter projected onto the atoms, Cu and chalcogen atoms were found to contribute more to the large total Grüneisen parameter. The obtained conclusions are determined by the following three comparisons: (1) The Cu–Se bond distance (2.5143 Å [59]) in BiCuSeO is longer than that in CuGaSe_2_ (2.385 Å [60]) in the case of the same tetrahedral coordination for Cu; (2) The Einstein temperature Θ_E_ for Cu and for the rattler atom of skutterudite, LaFe_4_Sb_12_ [61] is comparable; (3) The Cu–Se bond is soft and there is no suitable path for Cu mobility in layered structure. The Cu rattling mode [59] in BiCuSeO derived from the weak Cu–Se bonding was put forward. In summary, it can be seen that the presence of a localized low-energy vibrational mode on the Cu [59] contributes a great deal to the low thermal conductivity of BiCuSeO.

Generally, the contribution of high-frequency optical phonons to total lattice thermal conductivity is so small that it can be ignored. However, in the BiCuSeO system, Shao et al. [51] found that high-frequency (above 213 cm^−1^) optical phonons contributed more than one-third to the lattice thermal conductivity through first-principles calculations. Kumar et al. obtained a similar result that optical phonons can dramatically reduce lattice thermal conductivity κ_lat_ by a first principle phonon analysis [62]. Both reports pointed out that the contribution of optical phonons is considerable and should not be ignored, the results provide a promising route for further clarifying the origins of low thermal conductivity of BiCuSeO.

### 3.4. Microstructures Investigations on Heavy Ba-Doped BiCuSeO

**Figure 8:**
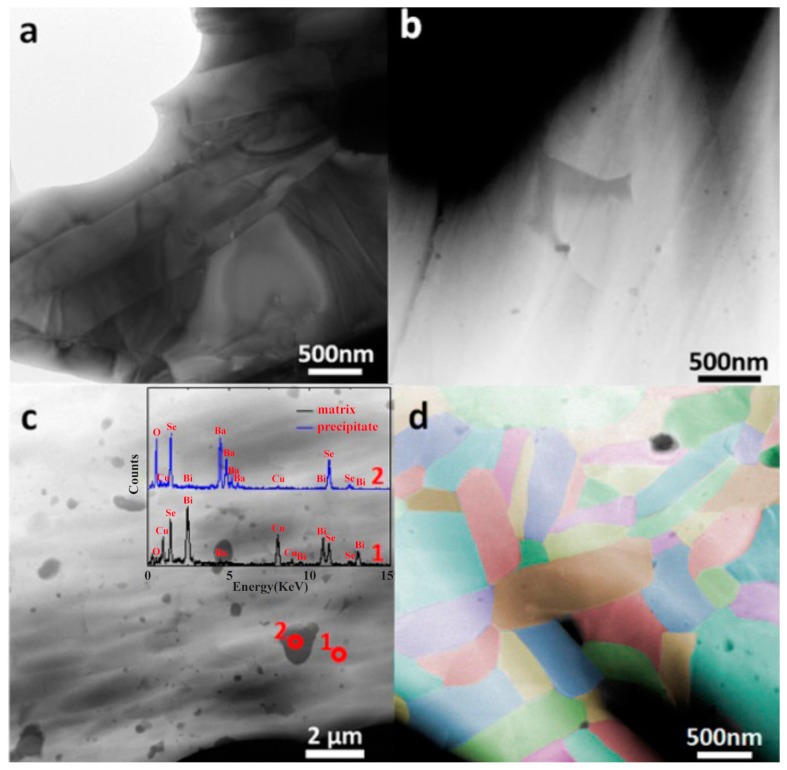
Low-magnification micrographs of (**a**) BiCuSeO in TEM mode; (**b**) Bi_0.95_Ba_0.05_CuSeO in STEM mode and (**c**) Bi_0.875_Ba_0.125_CuSeO in STEM mode; (**d**) Grains of Bi_0.875_Ba_0.125_CuSeO. Reproduced with permission from Reference [65]. Copyright 2016, Elsevier Science.

Extensive experiments confirmed that alkaline-earth metals doping is effective to enhance the thermoelectric performance of Bi_1−*x*_M*_x_*CuSeO (M = Mg, Ca, Sr, Ba) [1,2,4,6,30,63,64], among them the high-performance doped BiCuSeO systems exhibit the extremely low thermal conductivity. However, trying to explain origins of low thermal conductivity via investigating on the microstructures of these alkaline-earth-metal doped BiCuSeO remain largely unexplored. Feng et al. investigated the microstructures of Bi_0.875_Ba_0.125_CuSeO using scanning transmission electron microscopy (STEM) [65], and found a ubiquitous nano-scale BaSeO_3_ second phase in BiCuSeO matrix which played a positive role for further reduction of the thermal conductivity. The solubility limit of Ba in BiCuSeO was found to be less than 5% [65]. However, the highest *ZT* value was obtained in Bi_0.875_Ba_0.125_CuSeO, where as high as 12.5% Ba was dissolved in the matrix. The visual observation of the second phase BaSeO_3_ through STEM was a reasonable explanation of low thermal conductivity. As shown in Figure 8c, the dark precipitates are BaSeO_3_ which widely exist in the Bi_0.875_Ba_0.125_CuSeO, but there are no precipitates in Bi_0.95_Ba_0.05_CuSeO.

**Figure 9:**
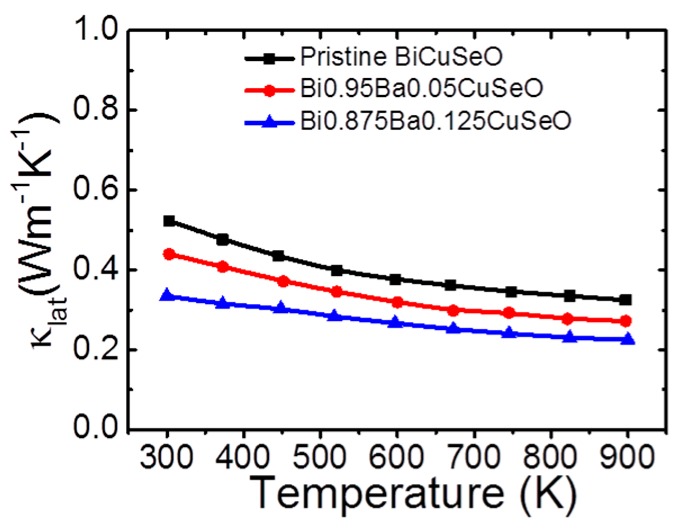
The lattice thermal conductivity of Bi_1−*x*_Ba*_x_*CuSeO (*x* = 0, 0.05, 0.125). Reproduced with permission from Reference [65]. Copyright 2016, Elsevier Science.

Benefitting from the all-scale phonons scattering from the interface of the layered structure, the Ba-Bi point defects and the BaSeO_3_ second phase, the lattice thermal conductivity of Bi_0.875_Ba_0.125_CuSeO is lower than that of pristine BiCuSeO and Bi_0.95_Ba_0.05_CuSeO in the entire temperature range, as shown in Figure 9.

## 4. Typical Examples to Enhance Thermoelectric Performance of BiCuSeO

### 4.1. Enhancing Electrical Conductivity by Modulation Doping

**Figure 10:**
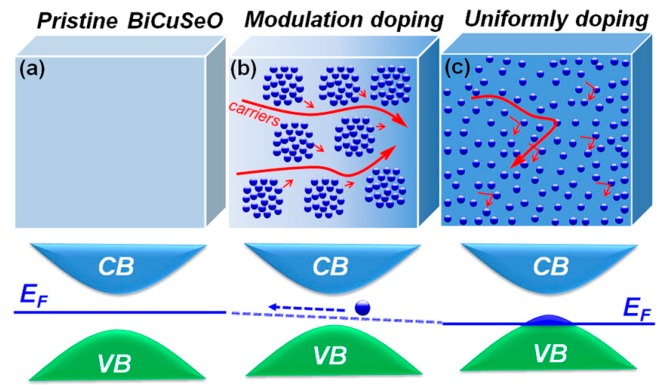
Three-dimensional schematic showing the band structures and Fermi energy levels for (**a**) the pristine BiCuSeO; (**b**) modulation doped Bi_0.875_Ba_0.125_CuSeO (50% BiCuSeO + 50% Bi_0.75_Ba_0.25_CuSeO); and (**c**) uniformly doped Bi_0.875_Ba_0.125_CuSeO. Reproduced (adapted) with permission from Reference [8]. Copyright 2014, American Chemical Society.

The electrical conductivity is determined by carrier concentration (n) and carrier mobility (μ). On the one hand, heavy doping can improve the carrier concentration significantly. On the other hand, the excessive dopants decrease the carrier mobility obviously because of the intensive ionized impurity scattering [66]. Therefore, the methods that enlarge the carrier mobility and maintain the carrier concentration can be expected to enhance the electrical transport properties. Pei et al. [8] proposed the utilization of 3D modulation doping in the synthesis of BiCuSeO. Even though modulation doping indeed works in a 2D material, however, further clarification is needed to confirm it exists in a 3D system. Figure 10a presents the pristine BiCuSeO which possesses relatively high carrier mobility (~22 cm^2^·V^−1^·s^−1^) and low carrier concentration (~1.1 × 10^18^ cm^−3^) [11]. In contrast, Figure 10c presents the uniformly heavy Ba-doped Bi_0.875_Ba_0.125_CuSeO with high carrier concentration (~1.2 × 10^21^ cm^−3^) and low carrier mobility (~2.1 cm^2^·V^−1^) [2] due to large ionized impurity scattering. Figure 10b presents the proposed mechanism of 3D modulation doping which is actually a two-phase composite with undoped BiCuSeO and heavy-doped Bi_0.875_Ba_0.125_CuSeO each accounting for 50 percent. Meanwhile, the Fermi level of modulation doping is higher than that of uniformly doping and lower than that of the undoped sample. There is a proceeding that holes flow from doped phase to undoped phase due to the successively small difference of chemical potential. Thus, the heavy doped phase sustains the high carrier concentration while the undoped phase provides a path that is easy to conduct, and both of these are responsible for the high electrical conductivity.

**Figure 11:**
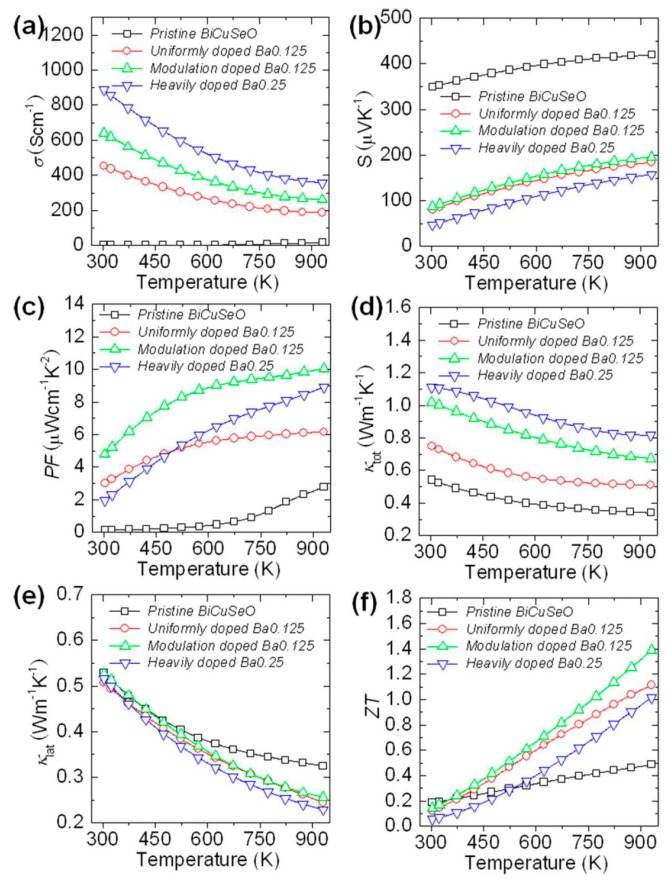
Thermoelectric properties of pristine BiCuSeO, uniformly doped Bi_0.875_Ba_0.125_CuSeO, modulation doping, and heavily Ba doped Bi_0.75_Ba_0.25_CuSeO. (**a**) The electrical conductivity; (**b**) the Seebeck coefficient; (**c**) the power factor; (**d**) total thermal conductivity; (**e**) lattice thermal conductivity; and (**f**) the figure of merit ZT. Reproduced (adapted) with permission from Reference [8]. Copyright 2014, American Chemical Society.

Figure 11 shows the thermoelectric properties of modulation doped Bi_0.875_Ba_0.125_CuSeO, undoped BiCuSeO, uniformly doped Bi_0.875_Ba_0.125_CuSeO and heavily doped Bi_0.75_Ba_0.25_CuSeO. Benefited from the high electrical concentration and mobility, the electrical conductivity of modulation doped Bi_0.875_Ba_0.125_CuSeO is higher than that of both undoped BiCuSeO and uniformly doped Bi_0.875_Ba_0.125_CuSeO at 300–923 K, as shown in Figure 11a. Besides, due to the relatively high Seebeck coefficient, the power factor of the modulation doped sample is much higher than the other three samples and reaches approximately 10 μW·cm^−1^·K^−2^ at 923 K, as shown in Figure 11b,c. Due to the high power factor and relatively low thermal conductivity (Figure 11d), the *ZT* value (Figure 11f) of the modulation doped sample has a great improvement, and the highest *ZT* ~ 1.4 is achieved at 923 K. These results indicate that the 3D modulation doping provides an effective method to improve the thermoelectric performance and affords an alternative reference for other thermoelectric systems.

### 4.2. Improving ZT Value of BiCuSeO by Synergetic Approaches

#### 4.2.1. Pb/Ca Dual-Doping

Lan et al. reported the electrical conductivity could be obviously enhanced through Pb doping at the Bi site. Besides, the Seebeck coefficient remains at a high level due to the increased effective mass. Both the higher electrical conductivity and comparable Seebeck coefficient lead to a larger power factor of Pb than that of other dopants [43]. Meanwhile, the Ca doping could reduce the lattice thermal conductivity effectively because of the large mass difference between Bi and Ca [6]. Therefore, Pb/Ca dual-doping simultaneously realized enhancing power factor and reducing thermal conductivity.

Liu et al. [10] introduced an all-scale structural optimization strategy, which is realized by dual-doped Ca and Pb at the Bi site to effectively enhance electrical conductivity and maintain Seebeck coefficient. Additionally, the dual-doping simultaneously optimized the microstructures at the atomic scale, nanoscale, and mesoscale, which could effectively reduce thermal conductivity. For the atomic scale, substitutions of Bi with Pb increased the carrier concentration and introduced point defects. For nanoscale and mesoscale, CaO_2_ nanoclusters and Bi-rich quantum dots besides the grain boundaries offset the increase of electrical conductivity originating from the increased carrier concentration. The novel dual-doping combined the advantages of these two dopants and achieved extremely good outcome as expected. 

**Figure 12:**
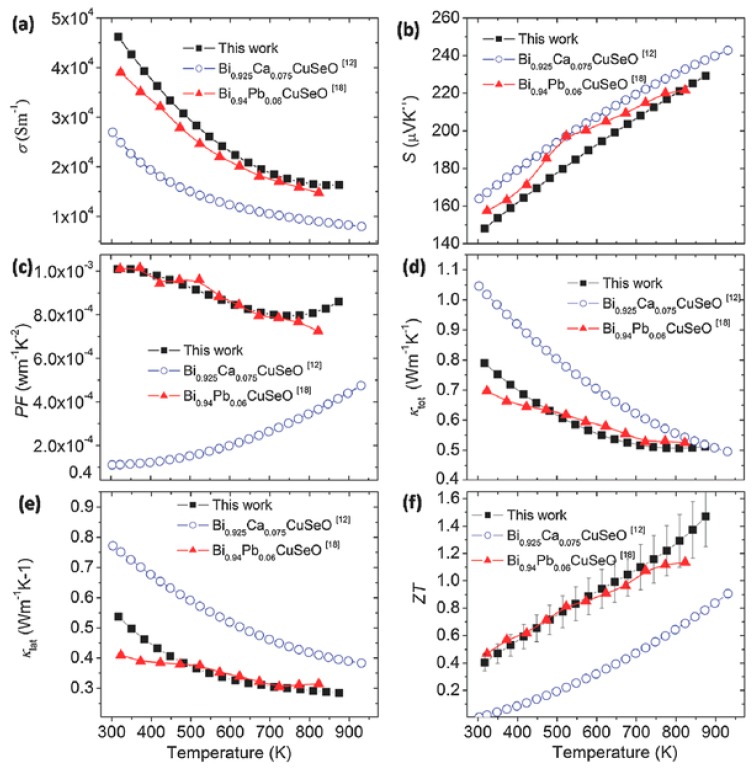
Thermoelectric properties of Pb and Ca dual-doping, Ca single doped (Bi_0.925_Ca_0.075_CuSeO) and Pb (Bi_0.94_Pb_0.06_CuSeO) single doped samples (**a**) the electrical conductivity; (**b**) the Seebeck coefficient; (**c**) the power factor; (**d**) total thermal conductivity; (**e**) lattice thermal conductivity; and (**f**) the figure of merit *ZT*. Reproduced with permission from Reference [10]. Copyright 2016, Wiley.

Figure 12 shows the effects of Pb/Ca dual-doping through comparing the best dual-doped Bi_0.88_Ca_0.06_Pb_0.06_CuSeO [10] with single doping Bi_0.925_Ca_0.75_CuSeO [6] and Bi_0.94_Pb_0.06_CuSeO [43] in TE properties. As shown in Figure 12a, the electrical conductivity of dual-doped Bi_0.88_Ca_0.06_Pb_0.06_CuSeO is much higher than that of Bi_0.925_Ca_0.075_CuSeO while slightly higher than that of Bi_0.94_Pb_0.06_CuSeO, which indicated in the positive role of Pb in regulating the electrical conductivity in the dual-doping. Meanwhile, due to a slightly difference in Seebeck coefficient (Figure 12b), the power factor (Figure 12c) of Bi_0.88_Ca_0.06_Pb_0.06_CuSeO is larger than that of Bi_0.925_Ca_0.075_CuSeO in all temperature ranges. The power factor of Bi_0.88_Ca_0.06_Pb_0.06_CuSeO below 700 K is comparable to Bi_0.94_Pb_0.06_CuSeO. While in a higher temperature range, the power factor of Bi_0.88_Ca_0.06_Pb_0.06_CuSeO is larger because of its higher Seebeck coefficient (Figure 12b). In addition, both the lattice thermal conductivity and the total thermal conductivity of Bi_0.88_Ca_0.06_Pb_0.06_CuSeO above 500 K are slightly lower than those of Bi_0.94_Pb_0.06_CuSeO (Figure 12d,e), which could be attributed to the all scale structural phonon scattering. Overall, the *ZT* value of dual-doped Bi_0.88_Ca_0.06_Pb_0.06_CuSeO reached a record high 1.5 at 873 K (Figure 12f).

#### 4.2.2. Bi/Cu Dual Vacancies

**Figure 13:**
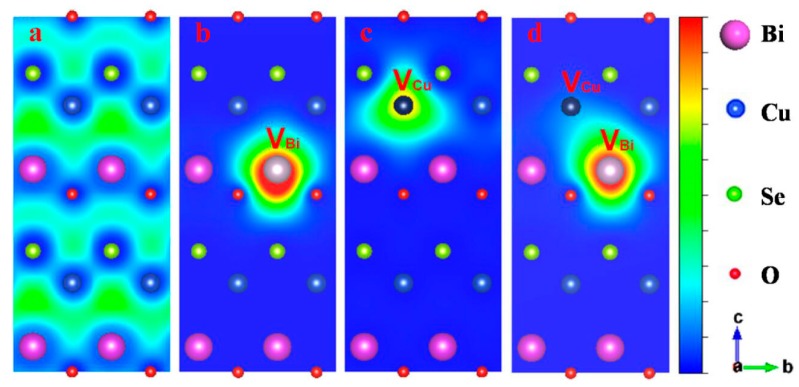
The projection of the positron density distribution for the (100) plane of (**a**) pure; (**b**) Bi vacancy; (**c**) Cu vacancy; and (**d**) Bi/Cu dual vacancies BiCuSeO samples. Reprinted (adapted) with permission from Reference [9]. Copyright 2015, American Chemical Society.

Li et al. [9] confirmed the existence of the inter-layer charge transmission mechanism between Bi vacancies and Cu vacancies through positron annihilation spectrometry (PAS). Served as the phonons scattering centers, vacancies in the lattice can reduce the thermal conductivity efficiently. However, vacancies also adversely affect the electrical conductivity due to the reception of electrons or holes. The introduction of the dual vacancies can reduce the thermal conductivity and improve the electrical conductivity at the same time. The reason is that when Cu vacancies and Bi vacancies are presented simultaneously, holes around the Bi vacancies’ centers will be transferred to the Cu vacancies’ centers. Because of the (Cu_2_Se_2_)^2−^ layers where Cu vacancies located being responsible for the carrier transmission in BiCuSeO, the increasing hole concentration can effectively enhance the electrical conductivity. Theoretically, the negatively charged centers of positive vacancies can annihilate positrons. As shown in Figure 13 b,c, the positrons are mostly trapped at the Bi vacancies and Cu vacancies in Bi_0.975_CuSeO and BiCu_0.975_SeO, respectively, while positrons are mostly distributed around Bi vacancies in Bi/Cu dual vacancy BiCuSeO (Figure 13d).

**Figure 14:**
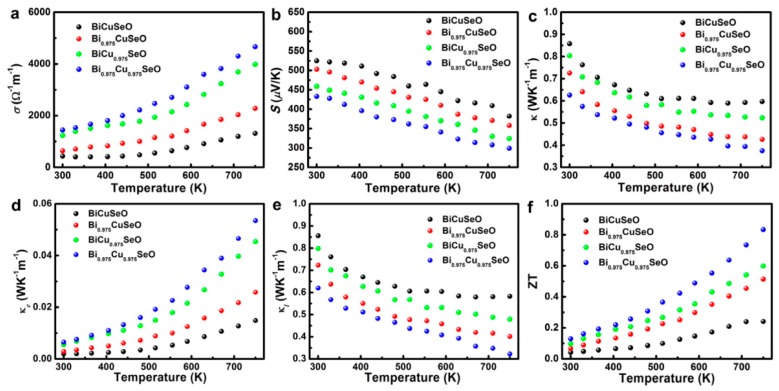
Thermoelectric properties of Bi_1−*x*_Cu_1−*y*_SeO samples, pristine BiCuSeO (black), Bi_0.975_CuSeO (red), BiCu_0.975_SeO (green), Bi_0.975_Cu_0.975_SeO (blue). (**a**) The electrical conductivity; (**b**) the Seebeck coefficient; (**c**) the total thermal conductivity; (**d**) electrical thermal conductivity; (**e**) lattice thermal conductivity; and (**f**) the figure of merit ZT. Reprinted (adapted) with permission from Reference [9]. Copyright 2015, American Chemical Society.

The projection of the positron density distribution proves the existence of carrier transmission from Bi vacancies to Cu vacancies in Bi/Cu dual vacancies BiCuSeO. It is easy to understand why the electrical conductivity of the dual vacancy sample is larger than that of monovacancy samples (Figure 14a). In addition, benefiting from the strong vacancy scattering in the whole temperature range (Figure 14e), the total thermal conductivity of dual vacancies sample is lower (Figure 14c) than that of monovacancy samples (Figure 14d). Hence, the *ZT* value of dual vacancy Bi_0.975_Cu_0.975_SeO is higher than those of monovacancy Bi_0.975_CuSeO and BiCu_0.975_SeO in the whole temperature range, resulting in a relatively high *ZT* of 0.84 was reached at 750 K for the dual vacancy sample (Figure 14f).

## 5. Summery and Perspective

In this review, we introduced the latest achievements and progress in BiCuSeO system, including enhancing thermoelectric properties through doping and compositing, developing less time-consuming methods, attempting to synthesize single crystals, exploring the origins of low thermal conductivity, and typical examples to enhance thermoelectric performance of BiCuSeO systems. Aside from the progress mentioned above, there is still room left for further focusing, such as the synthesis of BiCuSeO single crystal and a high performance *n*-type BiCuSeO, all of these are eagerly expected in the BiCuSeO system.

## References

[1] Zhao L.-D., Berardan D., Pei Y.L., Byl C., Pinsard-Gaudart L., Dragoe N. (2010). Bi_1−*x*_Sr*_x_*CuSeO oxyselenides as promising thermoelectric materials. Appl. Phys. Lett..

[2] Li J., Sui J., Pei Y., Barreteau C., Berardan D., Dragoe N., Cai W., He J., Zhao L.-D. (2012). A high thermoelectric figure of merit *ZT* > 1 in Ba heavily doped BiCuSeO oxyselenides. Energy Environ. Sci..

[3] Li F., Li J.-F., Zhao L.-D., Xiang K., Liu Y., Zhang B.-P., Lin Y.-H., Nan C.-W., Zhu H.-M. (2012). Polycrystalline BiCuSeO oxide as a potential thermoelectric material. Energy Environ. Sci..

[4] Li F., Wei T.-R., Kang F., Li J.-F. (2013). Enhanced thermoelectric performance of Ca-doped BiCuSeO in a wide temperature range. J. Mater. Chem. A.

[5] Pan L., Berardan D., Zhao L.-D., Barreteau C., Dragoe N. (2013). Influence of Pb doping on the electrical transport properties of BiCuSeO. Appl. Phys. Lett..

[6] Pei Y.-L., He J., Li J.-F., Li F., Liu Q., Pan W., Barreteau C., Berardan D., Dragoe N., Zhao L.-D. (2013). High thermoelectric performance of oxyselenides: Intrinsically low thermal conductivity of Ca-doped BiCuSeO. NPG Asia Mater..

[7] Sui J.H., Li J., He J.Q., Pei Y.L., Berardan D., Wu H.J., Dragoe N., Cai W., Zhao L.-D. (2013). Texturation boosts the thermoelectric performance of BiCuSeO oxyselenides. Energy Environ. Sci..

[8] Pei Y.L., Wu H., Wu D., Zheng F., He J. (2014). High thermoelectric performance realized in a BiCuSeO system by improving carrier mobility through 3D modulation doping. J. Am. Chem. Soc..

[9] Li Z., Xiao C., Fan S.J., Deng Y., Zhang W.S., Ye B.J., Xie Y. (2015). Dual vacancies: An effective strategy realizing synergistic optimization of thermoelectric property in BiCuSeO. J. Am. Chem. Soc..

[10] Liu Y., Zhao L.-D., Zhu Y., Liu Y., Li F., Yu M., Liu D.-B., Xu W., Lin Y.-H., Nan C.-W. (2016). Synergistically optimizing electrical and thermal transport properties of BiCuSeO via a dual-doping approach. Adv. Energy Mater..

[11] Zhao L.-D., He J., Berardan D., Lin Y., Li J.-F., Nan C.-W., Dragoe N. (2014). BiCuSeO oxyselenides: New promising thermoelectric materials. Energy Environ. Sci..

[12] Tan G., Shi F., Hao S., Zhao L.-D., Chi H., Zhang X., Uher C., Wolverton C., Dravid V.P., Kanatzidis M.G. (2016). Non-equilibrium processing leads to record high thermoelectric figure of merit in PbTe–SrTe. Nat. Commun..

[13] Heremans J.P., Jovovic V., Toberer E.S., Saramat A., Kurosaki K., Charoenphakdee A., Yamanaka S., Snyder G.J. (2008). Enhancement of thermoelectric efficiency in PbTe by distortion of the electronic density of states. Science.

[14] Zhao L.-D., Lo S.H., Zhang Y.S., Sun H., Tan G.J., Uher C., Wolverton C., Dravid V.P., Kanatzidis M.G. (2014). Ultralow thermal conductivity and high thermoelectric figure of merit in SnSe crystals. Nature.

[15] Zhao L.-D., Tan G.J., Hao S.Q., He J.Q., Pei Y.L., Chi H., Wang H., Gong S.K., Xu H.B., Dravid V.P. (2016). Ultrahigh power factor and thermoelectric performance in hole-doped single-crystal SnSe. Science.

[16] Tan G.J., Shi F.Y., Hao S.Q., Chi H., Zhao L.-D., Uher C., Wolverton C., Dravid V.P., Kanatzidis M.G. (2015). Codoping in SnTe: Enhancement of thermoelectric performance through synergy of resonance levels and band convergence. J. Am. Chem. Soc..

[17] Tan G.J., Shi F.Y., Hao S.Q., Chi H., Bailey T.P., Zhao L.-D., Uher C., Wolverton C., Dravid V.P., Kanatzidis M.G. (2015). Valence band modification and high thermoelectric performance in SnTe heavily alloyed with MnTe. J. Am. Chem. Soc..

[18] Tan G., Shi F., Doak J.W., Sun H., Zhao L.-D., Wang P., Uher C., Wolverton C., Dravid V.P., Kanatzidis M.G. (2015). Extraordinary role of hg in enhancing the thermoelectric performance of *p*-type SnTe. Energy Environ. Sci..

[19] Kim S.I., Lee K.H., Mun H.A., Kim H.S., Hwang S.W., Roh J.W., Yang D.J., Shin W.H., Li X.S., Lee Y.H. (2015). Dense dislocation arrays embedded in grain boundaries for high-performance bulk thermoelectrics. Science.

[20] Chung D.-Y., Hogan T., Brazis P., Rocci-Lane M., Kannewurf C., Bastea M., Uher C., Kanatzidis M.G. (2000). CsBi_4_Te_6_: A high-performance thermoelectric material for low-temperature applications. Science.

[21] Venkatasubramanian R., Siivola E., Colpitts T., O’Quinn B. (2001). Thin-film thermoelectric devices with high room-temperature figures of merit. Nature.

[22] Biswas K., Zhao L.-D., Kanatzidis M.G. (2012). Tellurium-free thermoelectric: The anisotropic *n*-type semiconductor Bi_2_S_3_. Adv. Energy Mater..

[23] Wang S., Tan G., Xie W., Zheng G., Li H., Yang J., Tang X. (2012). Enhanced thermoelectric properties of Bi_2_(Te_1−*x*_Se*_x_*)_3_-based compounds as *n*-type legs for low-temperature power generation. J. Mater. Chem..

[24] Shi X., Kong H., Li C.-P., Uher C., Yang J., Salvador J.R., Wang H., Chen L., Zhang W. (2008). Low thermal conductivity and high thermoelectric figure of merit in *n*-type Ba*_x_*Yb*_y_*Co_4_Sb_12_ double-filled skutterudites. Appl. Phys. Lett..

[25] Tan G., Liu W., Chi H., Su X., Wang S., Yan Y., Tang X., Wong-Ng W., Uher C. (2013). Realization of high thermoelectric performance in *p*-type unfilled ternary skutterudites FeSb_2+*x*_Te_1−*x*_ via band structure modification and significant point defect scattering. Acta Mater..

[26] Tan G., Zheng Y., Tang X. (2013). High thermoelectric performance of nonequilibrium synthesized CeFe_4_Sb_12_ composite with multi-scaled nanostructures. Appl. Phys. Lett..

[27] Tan G., Zheng Y., Yan Y., Tang X. (2014). Preparation and thermoelectric properties of *p*-type filled skutteruditesCe*_y_*Fe_4−*x*_Ni*_x_*Sb_12_. J. Alloys Compd..

[28] Tan G., Wang S., Li H., Yan Y., Tang X. (2012). Enhanced thermoelectric performance in zinc substituted *p*-type filled skutterudites CeFe_4−*x*_Zn*_x_*Sb_12_. J. Solid State Chem..

[29] Liu W.S., Zhang B.P., Li J.F., Zhao L.-D. (2007). Effects of SB compensation on microstructure, thermoelectric properties and point defect of CoSb_3_ compound. J. Phys. D Appl. Phys..

[30] Li J., Sui J., Barreteau C., Berardan D., Dragoe N., Cai W., Pei Y., Zhao L.-D. (2013). Thermoelectric properties of Mg doped *p*-type BiCuSeO oxyselenides. J. Alloys Compd..

[31] Liu Y.C., Zheng Y.H., Zhan B., Chen K., Butt S., Zhang B.P., Lin Y.H. (2015). Influence of Ag doping on thermoelectric properties of BiCuSeO. J. Eur. Ceram. Soc..

[32] Liu Y., Ding J., Xu B., Lan J., Zheng Y., Zhan B., Zhang B., Lin Y., Nan C. (2015). Enhanced thermoelectric performance of La-doped BiCuSeO by tuning band structure. Appl. Phys. Lett..

[33] Ren G.K., Butt S., Zeng C.C., Liu Y.C., Zhan B., Lan J.L., Lin Y.H., Nan C.W. (2015). Electrical and thermal transport behavior in Zn-doped BiCuSeO oxyselenides. J. Electron. Mater..

[34] Farooq M.U., Butt S., Gao K.W., Zhu Y.C., Sun X.G., Pang X.L., Khan S.U., Mohmed F., Mahmood A., Mahmood N. (2016). Cd-doping a facile approach for better thermoelectric transport properties of BiCuSeO oxyselenides. RSC Adv..

[35] Han M.K., Jin Y.S., Yu B.K., Choi W., You T.S., Kim S.J. (2016). Sulfur to oxygen substitution in BiCuSeO and its effect on the thermoelectric properties. J. Mater. Chem. A.

[36] Tan S.G., Lei H.C., Shao D.F., Lv H.Y., Lu W.J., Huang Y.N., Liu Y., Yuan B., Zu L., Kan X.C. (2014). Enhanced low temperature thermoelectric performance of Ag-doped BiCuSeO. Appl. Phys. Lett..

[37] Farooq M.U., Butt S., Gao K., Pang X.L., Sun X., Asfandiyar, Mohmed F., Ahmad A., Mahmood A., Mahmood N. (2017). Improved thermoelectric performance of BiCuSeO byAg substitution at cu site. J. Alloys Compd..

[38] Zhang M., Yang J., Jiang Q., Fu L., Xiao Y., Luo Y., Zhang D., Cheng Y., Zhou Z. (2015). Multi-role of sodium doping in BiCuSeO on high thermoelectric performance. J. Electron. Mater..

[39] Liu Y., Lan J., Zhang B., Lin Y., Nan C. (2016). Thermoelectric transport properties of BiCuSeO with embedded La_0.8_Sr_0.2_CoO_3_ nanoinclusions. Sci. China Technol. Sci..

[40] Liu Y.C., Zhou Y.M., Lan J.L., Zeng C.C., Zheng Y.H., Zhan B., Zhang B.P., Lin Y.H., Nan C.W. (2016). Enhanced thermoelectric performance of BiCuSeO composites with nanoinclusion of Cu selenides. J. Alloys Compd..

[41] Ren G.-K., Lan J.-l., Butt S., Ventura K.J., Lin Y.-H., Nan C.-W. (2015). Enhanced thermoelectric properties in Pb-doped BiCuSeO oxyselenides prepared by ultrafast synthesis. RSC Adv..

[42] Yang D., Su X., Yan Y., Hu T., Xie H., He J., Uher C., Kanatzidis M.G., Tang X. (2016). Manipulating the combustion wave during self-propagating synthesis for high thermoelectric performance of layered oxychalcogenide Bi_1–*x*_Pb*_x_*CuSeO. Chem. Mater..

[43] Lan J.L., Liu Y.C., Zhan B., Lin Y.H., Zhang B., Yuan X., Zhang W., Xu W., Nan C.W. (2013). Enhanced thermoelectric properties of Pb-doped BiCuSeO ceramics. Adv. Mater..

[44] Wang J.Y., Zhou Y.C., Lin Z.J. (2007). Mechanical properties and atomistic deformation mechanism of γ-Y_2_Si_2_O_7_ from first-principles investigations. Acta Mater..

[45] Dong S.T., Lv Y.Y., Zhang B.B., Zhang F., Yao S.H., Chen Y.B., Zhou J., Zhang S.T., Gu Z.B., Chen Y.F. (2015). Strong correlation of the growth mode and electrical properties of BiCuSeO single crystals with growth temperature. Crystengcomm.

[46] Wu X., Wang J.-L., Zhang H., Wang S., Zhai S., Li Y., Elhadj D., Fu G. (2015). Epitaxial growth and thermoelectric properties of *c*-axis oriented Bi_1−*x*_Pb*_x_*CuSeO single crystalline thin films. Crystengcomm.

[47] Samanta M., Guin S.N., Biswas K. (2017). Ultrathin few layer oxychalcogenide BiCuSeO nanosheets. Inorg. Chem. Front..

[48] Saha S.K., Dutta G. (2016). Elastic and thermal properties of the layered thermoelectrics BiCuSeO and LaOCuSe. Phys. Rev. B.

[49] Liu G., Sun H.Y., Zhou J., Li Q.F., Wan X.G. (2016). Thermal properties of layered oxychalcogenides BiCuOCh (Ch = S, Se, and Te): A first-principles calculation. J. Appl. Phys..

[50] Ji H.S., Togo A., Kaviany M., Tanaka I., Shim J.H. (2016). Low phonon conductivity of layered BiCuSO, BiCuSeO, and BiCuTeO from first principles. Phys. Rev. B.

[51] Shao H., Tan X., Liu G.-Q., Jiang J., Jiang H. (2016). A first-principles study on the phonon transport in layered bicuose. Sci. Rep..

[52] Hsiao C.-L., Qi X. (2016). The oxidation states of elements in pure and Ca-doped BiCuSeO thermoelectric oxides. Acta Mater..

[53] Chou T.-L., Tewari G.C., Chan T.-S., Hsu Y.-Y., Yamauchi H., Karppinen M. (2015). Exafs study of thermoelectric BiCuOSe: Effects of Cu vacancies. Solid State Commun..

[54] Berthebaud D., Guilmeau E., Lebedev O.I., Maignan A., Gamon J., Barboux P. (2016). The BiCu_1−*x*_OS oxysulfide: Cu deficiency and electronic properties. J. Solid State Chem..

[55] Li F., Wei T.R., Kang F.Y., Li J.F. (2014). Thermal stability and oxidation resistance of BiCuSeO based thermoelectric ceramics. J. Alloys Compd..

[56] Barreteau C., Berardan D., Dragoe N. (2015). Studies on the thermal stability of BiCuSeO. J. Solid State Chem..

[57] Ding J., Xu B., Lin Y., Nan C., Liu W. (2015). Lattice vibration modes of the layered material BiCuSeO and first principles study of its thermoelectric properties. New J. Phys..

[58] Saha S.K. (2015). Exploring the origin of ultralow thermal conductivity in layered BiOCuSe. Phys. Rev. B.

[59] Vaqueiro P., Al Orabi R.A., Luu S.D., Guelou G., Powell A.V., Smith R.I., Song J.P., Wee D., Fornari M. (2015). The role of Cu in the thermal conductivity of thermoelectric oxychalcogenides: Do lone pairs matter?. Phys. Chem. Chem. Phys..

[60] Abrahams S.C. (1974). Piezoelectric nonlinear optic CuGaSe_2_ and CdGeAs_2_: Crystal structure, chalcopyrite microhardness, and sublattice distortion. J. Chem. Phys..

[61] Sales B.C., Mandrus D.G., Chakoumakos B.C., Terry M.T. (2001). Chapter 1 use of atomic displacement parameters in thermoelectric materials research. Semiconductors and Semimetals.

[62] Kumar S., Schwingenschlogl U. (2016). Lattice thermal conductivity in layered BiCuSeO. Phys. Chem. Chem. Phys..

[63] Barreteau C., Berardan D., Amzallag E., Zhao L.-D., Dragoe N. (2012). Structural and electronic transport properties in Sr-doped BiCuSeO. Chem. Mat..

[64] Mizuno S., Ishizawa M., Fujishiro H., Naito T., Katsui H., Goto T. (2016). Ball milling effects for induced carriers and reduced grain size on thermoelectric properties in Bi_1−*x*_Sr*_x_*CuSeO (*x* = 0, 0.1). Jpn. J. Appl. Phys..

[65] Feng D., Zheng F.S., Wu D., Wu M.H., Li W., Huang L., Zhao L.-D., He J.Q. (2016). Investigation into the extremely low thermal conductivity in Ba heavily doped BiCuSeO. Nano Energy.

[66] Tan G.J., Zhao L.-D., Kanatzidis M.G. (2016). Rationally designing high-performance bulk thermoelectric materials. Chem. Rev..

